# Interleukin-6-Related Inflammatory Burden in Type 1 Diabetes: Evidence for Elevation with Suboptimal Glycemic Control

**DOI:** 10.3390/jcm14186511

**Published:** 2025-09-16

**Authors:** Theocharis Koufakis, Dimitrios Kouroupis, Areti Kourti, Katerina Thisiadou, Paraskevi Karalazou, Djordje S. Popovic, Dimitrios Patoulias, Giuseppe Maltese, Athina Pyrpasopoulou, Panagiotis Doukelis, Ioanna Zografou, Kalliopi Kotsa, Michael Doumas, Kali Makedou

**Affiliations:** 1 Second Propedeutic Department of Internal Medicine, Hippokration Hospital, School of Medicine, Aristotle University of Thessaloniki, 54642 Thessaloniki, Greece; dimcour841@gmail.com (D.K.); dipatoulias@gmail.com (D.P.); a.pyrpasopoulou@doctors.org.uk (A.P.); pitdukel@yahoo.gr (P.D.); ioannazo@yahoo.gr (I.Z.); doumasm@auth.gr (M.D.); 2 Laboratory of Biochemistry, AHEPA University Hospital, School of Medicine, Aristotle University of Thessaloniki, 54124 Thessaloniki, Greece; aretikourti@auth.gr (A.K.); thisiadou@yahoo.gr (K.T.); vivikarala@gmail.com (P.K.); kmakedou@auth.gr (K.M.); 3 Clinic for Endocrinology, Diabetes and Metabolic Disorders, Clinical Centre of Vojvodina, Medical Faculty, University of Novi Sad, 21000 Novi Sad, Serbia; djordje.popovic@mf.uns.ac.rs; 4 Department of Diabetes and Endocrinology, Epsom & St Helier University Hospitals, Epsom KT18 7EG, UK; giuseppe.maltese@kcl.ac.uk; 5 Unit for Metabolic Medicine, Cardiovascular Division, Faculty of Life Sciences & Medicine, King’s College, London WC2R 2LS, UK; 6 Division of Endocrinology and Metabolism and Diabetes Center, First Department of Internal Medicine, Medical School, Aristotle University of Thessaloniki, AHEPA University Hospital, 54636 Thessaloniki, Greece; kkalli@auth.gr

**Keywords:** biomarkers, glycemic control, inflammation, interleukin-6, type 1 diabetes, type 2 diabetes

## Abstract

**Background/Objectives**: Inflammation is a hallmark of diabetes, with interleukin-6 (IL-6) emerging as a key mediator linking immune activation with metabolic regulation. Although IL-6 has been studied in both type 1 (T1D) and type 2 diabetes (T2D), its relationship with glycemic control across diabetes subtypes remains unexplored. **Methods**: We conducted a cross-sectional pilot study including 82 participants divided into the following subgroups: healthy controls (*n* = 14), individuals with T1D [*n* = 11 with glycated hemoglobin (HbA1c) < 7%; *n* = 11 with HbA1c ≥ 7%] and T2D (*n* = 21 with HbA1c < 7%; *n* = 25 with HbA1c ≥ 7%). Demographic, anthropometric, and laboratory parameters were collected. Group comparisons were performed, adjusted for age and body mass index (BMI) to account for significant demographic differences between groups. Correlations between IL-6, high-sensitivity C-reactive protein (hs-CRP), ferritin, and presepsin were evaluated using Spearman’s rank correlation. **Results**: IL-6 levels were approximately four-fold higher in T1D individuals with HbA1c ≥ 7% compared with controls [fold-change 4.06 (95% CI: 1.36–12.1), *p* = 0.013], with optimally managed T1D showing a non-significant trend (*p* = 0.079). No significant differences were observed in T2D groups. Advancing age demonstrated a borderline association with IL-6 (*p* = 0.068), whereas BMI was not significantly related. IL-6 correlated positively with hs-CRP (ρ = 0.463, *p* < 0.001), but not with ferritin or presepsin. **Conclusions**: IL-6 concentrations were significantly elevated in individuals with suboptimally managed T1D compared with controls, independent of age and BMI, suggesting that poor metabolic control amplifies systemic inflammation in autoimmune diabetes. These findings support IL-6 as a biomarker of inflammatory burden in T1D and provide a rationale for larger, longitudinal studies to determine its clinical utility.

## 1. Introduction

Type 1 diabetes mellitus (T1D) and type 2 diabetes mellitus (T2D) develop through distinct mechanisms but both result in persistent hyperglycemia. T1D stems from autoimmune destruction of pancreatic β-cells, leading to absolute insulin deficiency and lifelong reliance on insulin therapy [1]. T2D is characterized mainly by peripheral insulin resistance, gradually compounded by β-cell dysfunction and impaired insulin secretion [2]. Clinically, T1D usually presents earlier in life and often in individuals without obesity, whereas T2D is typically diagnosed later and is strongly linked to obesity, sedentary lifestyle, and metabolic syndrome. Despite these differences, both forms of diabetes converge on chronic hyperglycemia, which drives systemic metabolic disturbances and vascular complications [3].

T1D arises from a complex interplay of genetic, environmental, and immunological factors. Genetic susceptibility is strongly linked to HLA class II alleles, which modulate the adaptive immune response [4]. Environmental exposures, such as viral infections, early-life diet, and gut microbiome alterations, have also been implicated as triggers in genetically predisposed individuals [5]. The disease typically develops in childhood or adolescence but can occur at any age, reflecting heterogeneity in pathogenesis. Long-term complications include microvascular outcomes such as retinopathy, nephropathy, and neuropathy, as well as macrovascular disease, which remains a leading cause of morbidity and mortality in T1D [6]. These complications are closely linked to chronic hyperglycemia and inflammation, underscoring the need for biomarkers that capture inflammatory burden.

Inflammation plays a central role in the pathogenesis and progression of both forms of diabetes. In T2D, adipose tissue expansion and altered metabolic signaling promote the secretion of inflammatory mediators, which reinforce insulin resistance [7]. In T1D, the autoimmune attack on pancreatic β-cells is the initiating event, but systemic inflammatory markers are also increased and may contribute to complications [8]. Across both types of diabetes, elevated cytokines and acute-phase proteins highlight the importance of immune–metabolic interactions as a shared feature despite different underlying mechanisms [9].

Ferritin is an acute-phase reactant that reflects both iron metabolism and systemic inflammatory activity, and elevated levels have been reported in individuals with diabetes and metabolic syndrome [10]. Presepsin, a soluble CD14 subtype, has been proposed as a link between inflammation and innate immune responses; in diabetes, its concentrations have been associated with glycemic variability and the burden of complications [11]. High-sensitivity C-reactive protein (hs-CRP) is a widely established biomarker of systemic inflammation and a downstream effector of IL-6 signaling, with numerous studies linking it to poor glycemic control and cardiovascular risk in both T1D and T2D [12]. 

Among inflammatory mediators, interleukins are of particular interest because of their diverse roles in regulating immune activity and metabolism. Pro-inflammatory cytokines such as interleukin (IL)-1β and tumor necrosis factor-α impair β-cell survival and promote insulin resistance, whereas other interleukins, like IL-10, counterbalance these effects through anti-inflammatory pathways [13]. IL-6 is especially noteworthy because of its dual functions. On the one hand, it promotes acute inflammatory responses and immune activation; on the other, it influences glucose metabolism by modulating hepatic glucose output, lipid breakdown, and adipose tissue activity [14]. In T1D, several mechanisms may contribute to increased IL-6 concentrations. Persistent autoimmune activity, even beyond the initial phase of β-cell destruction, promotes ongoing cytokine release [15]. Chronic hyperglycemia further amplifies oxidative stress and immune cell activation, both of which can stimulate IL-6 production [16]. In addition, greater glycemic variability and hypoglycemia burden in T1D have been linked to systemic inflammatory responses that may upregulate IL-6 [17]. Together, these mechanisms suggest that IL-6 may reflect the interplay between autoimmunity, metabolic dysregulation, and inflammation in T1D. Because of its central role at the interface of immunity and metabolism, IL-6 was selected as the primary biomarker of interest in this study.

Despite this evidence, the relationship between IL-6 and glycemic control in diabetes remains poorly defined. Most studies have either examined diabetes as a single entity or focused exclusively on T2D. Data in T1D, especially when stratifying by glycemic status, are scarce. Clarifying how IL-6 relates to metabolic control in diabetes, particularly in T1D, may help identify patients at higher inflammatory risk and guide its use as a biomarker of disease burden. Determining whether IL-6 elevation is specific to autoimmune diabetes or a more general feature of poor glycemic control carries implications for both risk stratification and the development of targeted interventions. This gap motivated the present study, which aimed to compare IL-6 concentrations across individuals with T1D, T2D, and normoglycemic controls. A secondary aim was to explore correlations between IL-6 and additional inflammatory markers.

## 2. Materials and Methods

### 2.1. Study Design

This work was designed as a cross-sectional pilot study and was carried out at the Aristotle University of Thessaloniki, Greece, from January to May 2024. Eligible participants were adults (≥18 years) with a confirmed diagnosis of T1D or T2D defined according to the American Diabetes Association criteria [18]. Exclusion criteria were: (i) acute infection, surgery, or severe inflammatory condition within the last three months; (ii) recent diabetic ketoacidosis or hyperosmolar hyperglycemic state; (iii) treatment with medications known to alter body weight or glucose regulation other than antidiabetics, statins, or antihypertensives (e.g., corticosteroids, antipsychotics); (iv) advanced liver disease; (v) presence of autoimmune disease or malignancy; and (vi) established microvascular (including diabetic nephropathy) or macrovascular complications, since are known to strongly affect inflammatory status.

**Figure 1:**
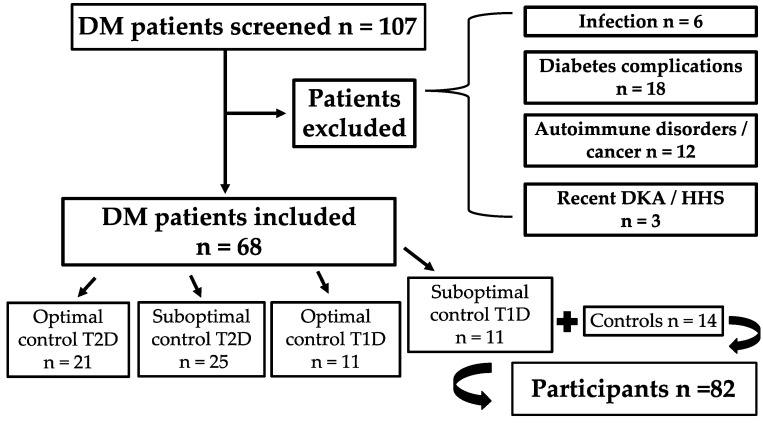
Flowchart of participant selection and stratification. Abbreviations: DM: diabetes mellitus; T1D: type 1 diabetes; T2D: type 2 diabetes; DKA: diabetic ketoacidosis; HHS: hyperosmolar hyperglycemic state.

Healthy participants were recruited among hospital staff. They had no personal history of diabetes or prediabetes, and no acute or chronic inflammatory or systemic illness. Individuals with diabetes were stratified by glycemic status into two subgroups: optimal glycemic control [glycated hemoglobin (HbA1c) < 7%] and suboptimal glycemic control (HbA1c ≥ 7%). A flowchart illustrating participant screening, application of exclusion criteria, and stratification into study subgroups is provided in Figure 1.

### 2.2. Data Collection

Each participant attended a single structured visit, during which demographic, anthropometric, and laboratory data were obtained. Weight was recorded to the nearest 0.1 kg on a calibrated digital scale, with subjects lightly clothed and without shoes. Body mass index (BMI) was calculated as weight divided by height squared (kg/m^2^). Waist circumference (WC) was assessed midway between the inferior margin of the rib cage and the iliac crest with an anthropometric tape. All measurements were performed by one investigator and independently confirmed by another to ensure accuracy.

Blood samples were obtained by venipuncture after a 12 h overnight fast. Samples were promptly processed, and serum was stored at –20 °C until analysis. Electrochemiluminescence immunoassays performed on the ELECSYS analyzer were used for the measurement of ferritin and IL-6, while hs-CRP was determined by a particle-enhanced immunoturbidimetric assay on the COBAS Pure analyzer; both systems were supplied by Roche Diagnostics (Mannheim, Germany). Serum presepsin concentrations were quantified with a commercially available ELISA kit (Wuhan Fine Biotech Co., Ltd., Wuhan, China). All analyses were performed in the same central laboratory. Laboratory analyses were conducted by investigators blinded to participant group allocation. Samples were coded and processed in random order to minimize measurement bias.

### 2.3. Statistical Analysis

Continuous variables were summarized as means with standard deviations, while categorical data were presented as counts and percentages. Between-group comparisons of age and BMI were performed using one-way analysis of variance (ANOVA) with Tukey’s honestly significant difference test for post hoc pairwise testing. IL-6 values were examined for distributional properties and showed a markedly right-skewed distribution, with a considerable proportion of results recorded at the lower assay detection limit of 1.5 pg/mL. The exact values of these measurements were unknown but known to be below the cutoff; therefore, they were treated as left-censored data. Group differences in IL-6 were first assessed using analysis of covariance (ANCOVA), adjusting for age and BMI. To account for the skewed distribution and censoring at the detection threshold, Tobit regression was then applied to log-transformed IL-6 concentrations. This method allowed estimation of adjusted central tendencies, predicted probabilities of exceeding the detection limit, and fold-changes relative to controls, with *p*-values derived from Wald tests. As a sensitivity analysis, models were additionally adjusted for WC to account for central adiposity. Given this distributional profile, IL-6 values are reported only in model-adjusted form, rather than as raw medians. Correlations between IL-6, presepsin, ferritin, and hs-CRP were examined using Spearman’s rank correlation coefficients (ρ) with corresponding *p*-values. All analyses were conducted using Python (statsmodels v0.14). Statistical significance was set at a two-sided *p* < 0.05.

### 2.4. Ethical Aspects

This study was conducted according to international ethical standards. Written informed consent was obtained from all participants before enrolment. The research protocol was reviewed and approved by the Scientific Council of Hippokration General Hospital of Thessaloniki (approval No. 47699/23-10-2023). Participant confidentiality was ensured throughout the study.

## 3. Results

### 3.1. Study Population

A total of 82 participants were included and divided into the following subgroups: controls (*n* = 14), individuals with T1D and HbA1c < 7% (*n* = 11), T1D and HbA1c ≥ 7% (*n* = 11), T2D and HbA1c < 7% (*n* = 21), and T2D with HbA1c ≥ 7% (*n* = 25). Age differed significantly across groups (ANOVA *F*(4, 77) = 17.1, *p* < 0.001), with participants with T2D being older on average than those with T1D, while controls were of intermediate age. As expected, BMI also differed significantly (ANOVA *F*(4, 77) = 5.61, *p* < 0.001), with higher BMI values observed in the T2D groups compared with controls and T1D. However, pairwise post hoc comparisons using Tukey’s test did not reach significance after adjustment for multiple testing. Group-specific demographic data are presented in Table 1.

### 3.2. Interleukin-6 Concentrations Across Groups

IL-6 values showed a markedly skewed distribution, with many measurements at the lower assay detection limit of 1.5 pg/mL. As a result, raw descriptive medians are not presented; instead, regression-adjusted estimates from Tobit models are reported, as these appropriately account for skewness and censoring. In the ANCOVA model adjusting for age and BMI, no statistically significant overall differences in IL-6 were detected among the five groups (*p* = 0.212). Individuals with optimally controlled T1D exhibited the highest adjusted concentrations, approximately double those of controls, although this trend was not statistically significant after multiple testing correction. However, the Tobit regression analysis demonstrated a significant elevation of IL-6 in a group of individuals with suboptimally controlled T1D compared with controls (fold-change ≈ 4.1, *p* = 0.013). A similar, non-significant trend was observed for optimally managed individuals with T1D (fold-change ≈ 2.4, *p* = 0.079). No significant differences were found for T2D groups versus controls. Increasing age was associated with higher IL-6 concentrations (*p* = 0.068), while BMI was not significant. When WC was included alongside BMI as a covariate, evidence of multicollinearity was observed, yet the overall pattern of IL-6 associations across groups remained consistent. Model-derived estimates are shown in Table 2.

### 3.3. Correlations Between Biomarkers

Across the overall cohort, IL-6 was moderately correlated with hs-CRP (ρ = 0.463, *p* < 0.001). IL-6 showed weak, non-significant associations with ferritin (ρ = –0.227, *p* = 0.060) and presepsin (ρ = 0.119, *p* = 0.332). Ferritin, presepsin, and hs-CRP were not significantly correlated with each other. Stratified analyses by group yielded similar patterns, with positive IL-6–hs-CRP correlations most evident in individuals with T1D and suboptimal control, though subgroup results did not consistently achieve statistical significance, likely due to limited sample sizes (Table 3).

## 4. Discussion

In this pilot study, we compared circulating IL-6 levels across groups of individuals with T1D, T2D and healthy participants, with further stratification by glycemic status. The key finding was that IL-6 levels were markedly higher in participants with suboptimally managed T1D, whereas no significant differences were observed in T2D. To our knowledge, this is the first investigation to directly compare IL-6 across both diabetes types while accounting for glycemic state, revealing a novel link between inflammatory burden and suboptimal glucose control in T1D.

The role of IL-6 in T1D appears to be multifaceted, with both detrimental and potentially beneficial effects. On the detrimental side, IL-6 is a key upstream driver of hepatic CRP synthesis and has been linked to endothelial dysfunction, insulin resistance, and systemic inflammatory activation, all of which may accelerate vascular complications in T1D [19]. Persistent IL-6 elevation may also reflect chronic immune activation, which can worsen metabolic instability and long-term outcomes. At the same time, IL-6 exerts beneficial effects under certain conditions. It plays an important role in exercise physiology, where transient increases promote glucose uptake and lipid oxidation, potentially improving metabolic flexibility [20]. Moreover, IL-6 signaling can stimulate anti-inflammatory mediators such as IL-10, highlighting its context-dependent effects [21]. Thus, in T1D, IL-6 may act both as a marker of detrimental immune–metabolic stress and, under specific conditions, as a mediator of adaptive responses. This duality complicates interpretation but underscores the importance of studying IL-6 in carefully defined clinical contexts.

Previous studies have consistently demonstrated that individuals with T1D exhibit higher circulating IL-6 levels compared with healthy controls, suggesting an underlying pro-inflammatory milieu in this population [22]. A meta-analysis of case–control studies confirmed significantly elevated IL-6 in T1D irrespective of age, ethnicity, or duration of the condition [23]. However, few investigations have explored whether the degree of glycemic control influences IL-6 concentrations. Our work extends this knowledge by showing that IL-6 was particularly elevated in the uncontrolled T1D group, with levels approximately four-fold higher than in controls after adjustment for age and BMI. The observed trend toward increased IL-6 even in controlled T1D suggests that systemic inflammation may be present in T1D regardless of metabolic regulation, but appears to be accentuated in poor glycemic states.

Our current observation that IL-6 is highest in uncontrolled T1D than controls complements but also partly contrasts with our recent continuous glucose monitoring (CGM)-based study in adults with T1D [24]. In that analysis, IL-6 correlated positively with time below range but negatively with time above range (TAR), suggesting that hypoglycemia may be a stronger acute driver of IL-6 release than hyperglycemia. In the present study, groupwise comparisons were based on HbA1c, which reflects long-term average glycemia but does not capture short-term variability. The finding that IL-6 was highest in participants with poorly regulated T1D may therefore reflect not only greater TAR but also increased glycemic instability and hypoglycemia burden, both of which are typically more pronounced in this group [25,26]. An additional explanation is that chronic poor control may induce inflammatory signaling through mechanisms such as oxidative stress and advanced glycation [27], which are not directly reflected by CGM-derived TAR. Taken together, these findings suggest that IL-6 is influenced by both the quality and the stability of glucose regulation.

In contrast to T1D, IL-6 concentrations in participants with T2D did not differ significantly from controls in our study. This finding diverges from several prior reports that linked T2D and obesity with elevated IL-6, typically attributed to adipose tissue–driven inflammation [28,29,30]. One explanation for the discrepancy may be methodological, as our relatively small sample size and the heterogeneity of treatments in T2D could have attenuated group differences. Medications commonly prescribed for T2D, including glucagon-like peptide-1 receptor agonists, sodium-glucose cotransporter 2 inhibitors, and pioglitazone, have well-described anti-inflammatory effects and may have lowered circulating IL-6 in treated individuals [31]. In contrast, insulin remains the sole therapy in T1D, which removes this confounding factor. Moreover, the autoimmune background of T1D maintains a state of chronic immune activation, even beyond the period of acute β-cell destruction [32]. In this setting, poor glycemic control may further amplify immune cell activity, oxidative stress, and cytokine production, resulting in a disproportionate rise in IL-6 [33,34,35]. Taken together, these findings suggest that IL-6 elevation is not simply a reflection of hyperglycemia but rather reflects the interaction between poor metabolic control and underlying autoimmunity in T1D. In T2D, obesity-related inflammation and the modifying effects of diverse therapies may mask such associations, leading to the absence of significant differences in IL-6 concentrations in our cohort.

IL-6 showed a robust positive correlation with hs-CRP in the overall study sample. IL-6 and CRP are closely interconnected in T1D. IL-6 acts as a primary upstream regulator of hepatic CRP synthesis [36], and this biological link explains the positive correlation observed in our study. Elevated CRP levels have been consistently associated with increased cardiovascular risk in both the general population and in individuals with diabetes [37]. In T1D specifically, higher CRP concentrations have been linked to endothelial dysfunction, microvascular complications, and accelerated atherosclerosis [38]. IL-6 therefore serves as both a trigger of systemic inflammation and a potential predictor of downstream CRP responses. The combined assessment of IL-6 and CRP may provide complementary prognostic information, capturing upstream immune activation (IL-6) and downstream hepatic inflammatory signaling (CRP). This dual biomarker strategy may be particularly relevant for stratifying vascular risk in patients with poorly controlled T1D.

In contrast, IL-6 associations with ferritin and presepsin were weak and did not reach statistical significance, suggesting that these markers may be influenced by additional regulatory pathways beyond systemic inflammation alone. Presepsin, in particular, has been implicated in the interaction between innate immunity and gut microbiota, suggesting a potential link between inflammation and dysbiosis [39]. In our previous studies, presepsin correlated with glycemic variability in T1D and with the degree of glycemic control in T2D [40,41]. Its lack of association with IL-6 in the present work may therefore reflect the multifaceted nature of its biology, with contributions from microbial translocation, metabolic stress, and immune activation that differ across diabetes phenotypes.

The therapeutic implications of targeting IL-6 signaling in T1D warrant consideration. IL-6 receptor antagonists such as tocilizumab and sarilumab are already approved for the treatment of rheumatoid arthritis and other autoimmune conditions, and pilot studies have begun exploring their potential in T1D [42]. Early data suggest that IL-6 inhibition may help preserve residual β-cell function and attenuate systemic inflammation, though results remain preliminary [43]. From a clinical perspective, our findings of elevated IL-6 in uncontrolled T1D support the rationale for such interventions. However, the dual nature of IL-6 biology—detrimental in chronic inflammation but beneficial in metabolic adaptation—underscores the need for careful patient selection and long-term evaluation of safety and efficacy. Larger randomized studies are required before IL-6 inhibition can be recommended as a therapeutic approach in T1D.

The strengths of this study include the simultaneous assessment of multiple inflammatory biomarkers, the stratification of both T1D and T2D participants by glycemic control, and the use of Tobit regression to account for censoring at the lower detection limit of IL-6. This multifaceted approach enhanced the robustness of our findings, particularly in highlighting the elevation of IL-6 in suboptimally managed T1D. Another strength lies in the inclusion of both autoimmune and insulin-resistant diabetes, allowing for a direct comparison of inflammatory patterns across distinct pathophysiological entities. By adjusting for key confounders such as age and BMI, we were able to partially disentangle the effects of glycemic control from demographic or anthropometric variability. Moreover, the careful application of exclusion criteria minimized the influence of comorbidities and overt infections, thereby increasing the internal validity of the results.

Nevertheless, several limitations should be acknowledged. First, the cross-sectional design precludes causal inference between glycemic control and inflammatory activity. The associations reported here should therefore be interpreted as hypothesis-generating rather than definitive. Second, the relatively small subgroup sizes may have limited statistical power to detect subtle differences, particularly in T2D, and may have contributed to the wide confidence intervals. Third, the absence of longitudinal follow-up prevents us from determining whether improvement in glycemic control translates into reductions in IL-6 levels over time. Fourth, although we adjusted for age and BMI, additional unmeasured factors—including dietary intake, physical activity, medication adherence, and subclinical infections—could have influenced biomarker levels. Furthermore, the heterogeneity of pharmacological regimens in T2D complicates interpretation, as several widely used agents possess anti-inflammatory properties that may have masked associations. Finally, as this was a single-center pilot study, the generalizability of the findings may be limited, and replication in larger, more diverse cohorts will be essential.

In conclusion, our study demonstrates that IL-6 concentrations are significantly elevated in individuals with suboptimally managed T1D compared with controls after adjustment for age and anthropometry, while no differences were observed in T2D. From a clinical perspective, these findings suggest that IL-6 may serve as a biomarker of inflammatory burden in T1D, particularly in the context of poor glycemic control. Elevated IL-6 could potentially identify patients at higher inflammatory risk and complement established measures of metabolic status such as HbA1c and CGM metrics.

Looking forward, the clinical application of IL-6 measurement in diabetes management requires further exploration. Longitudinal studies should examine whether dynamic changes in IL-6 mirror fluctuations in glycemic control, and whether targeted interventions—either lifestyle-based or pharmacological—are capable of modulating IL-6 levels in parallel with improved outcomes. Moreover, the dual role of IL-6, encompassing both detrimental and potentially adaptive functions, calls for careful study designs that can distinguish between transient, beneficial IL-6 elevations (e.g., during exercise) and chronic, harmful increases linked to autoimmune activation.

Future work should also investigate IL-6 in combination with other biomarkers, such as hs-CRP, presepsin, and ferritin, to determine whether multimarker panels provide more robust prognostic information than single analytes. In addition, the potential role of IL-6 inhibitors, already in clinical use for other autoimmune disorders, deserves further study in T1D, particularly in patients with high inflammatory burden and poor glycemic control. Ultimately, integrating inflammatory profiling into clinical practice could open the way to more personalized approaches in diabetes care, where risk stratification and therapeutic monitoring are informed not only by glycemic indices but also by the underlying immune–metabolic state.

## Figures and Tables

**Table 1:** Demographic characteristics of the study population.

Group	N	Age, Mean (SD), Years	BMI, Mean (SD), kg/m^2^
Control	14	54.1 (7.8)	24.5 (2.5)
Type 2 with suboptimal control	25	63.9 (14.1)	32.3 (7.3)
Type 2 with optimal control	21	60.4 (10.4)	30.1 (4.1)
Type 1 with suboptimal control	11	30.7 (11.9)	25.7 (5.5)
Type 1 with optimal control	11	45.8 (10.4)	28.0 (3.5)
Overall	82	52.6 (16.6)	29.0 (5.9)

SD: standard deviation; BMI: Body mass index.

**Table 2:** Tobit regression–adjusted IL-6 results (adjusted for age and body mass index).

Group	Adjusted IL-6, pg/mL	Predicted Probability (IL-6 > 1.5 pg/mL)	Fold-Change vs. Control (95% CI)	*p* Value
Control	1.79	31%	Reference	–
Type 1 with optimal control	2.72	67%	2.35 (0.91–6.06)	0.079
Type 1 with suboptimal control	4.14	85%	4.06 (1.36–12.1)	0.013
Type 2 with optimal control	2.08	47%	1.46 (0.60–3.55)	0.401
Type 2 with suboptimal control	1.99	42%	1.32 (0.53–3.28)	0.562

IL-6: Interleukin-6; CI: Confidence interval.

**Table 3:** Correlation matrix of biomarkers in the overall study population (Spearman’s ρ with *p*-values).

	IL-6	hs-CRP	Ferritin	Presepsin
IL-6	1.000 (*p* = 1.0)	0.463 (*p* < 0.001)	–0.227 (*p* = 0.060)	0.119 (*p* = 0.332)
hs-CRP	0.463 (*p* < 0.001)	1.000 (*p* = 1.0)	–0.157 (*p* = 0.198)	0.077 (*p* = 0.522)
Ferritin	–0.227 (*p* = 0.060)	–0.157 (*p* = 0.198)	1.000 (*p* = 1.0)	0.027 (*p* = 0.828)
Presepsin	0.119 (*p* = 0.332)	0.077 (*p* = 0.522)	0.027 (*p* = 0.828)	1.000 (*p* = 1.0)

IL-6: interleukin 6; hs-CRP: high-sensitivity C-reactive protein.

## Data Availability

The data presented in the study are available on request from the corresponding author. The data are not publicly available due to privacy restrictions of the Greek National Health System.
